# Unconventional Therapies in Periprosthetic Joint Infections: Prevention and Treatment: A Narrative Review

**DOI:** 10.3390/jcm14082610

**Published:** 2025-04-10

**Authors:** Daniyil Semeshchenko, Pablo A. Slullitel, Alicia Farinati, Agustin F. Albani-Forneris, Nicolas S. Piuzzi, Martin A. Buttaro

**Affiliations:** 1 ‘Sir John Charnley’ Hip Surgery Unit, Institute of Orthopaedics ‘Carlos E. Ottolenghi’, Italian Hospital of Buenos Aires, 4190 Perón St., Buenos Aires C1199ABH, Argentina; 2 Institute of Medical and Health Sciences Research (IIMCS), Faculty of Medicine, Salvador University, 1601 Córdoba Av., Buenos Aires C1055AAG, Argentina; 3 Department of Orthopaedic Surgery Cleveland, Cleveland Clinic Foundation, Cleveland, OH 44195, USA

**Keywords:** biofilm, periprosthetic joint infection, unconventional treatment, new strategies, infection prevention

## Abstract

Background: as the demand for total joint arthroplasty continues to grow each year, the healthcare burden is expected to increase due to periprosthetic joint infection (PJI). This review article aims to highlight the significance of biofilms in the pathogenesis of PJI and introduce alternative therapies that prevent bacterial adhesion to implants or enhance their eradication when infection occurs. Search strategy: we conducted a bibliographic search in PubMed using the following MeSH terms as follows: “no antibiotic treatment of PJI”, “bacterial biofilm eradication agents”, and “unconventional prevention of PJI”, among others. Most important results: after an initial analysis of the literature, we selected the most significant topics on novel PJI treatment methods and prevention strategies. A second PubMed search highlighted the following therapeutic modalities: the application of hydrogels on implant surfaces, the use of phage therapy, lysostaphin and antimicrobial peptides, the implementation of two-stage debridement, irrigation, implant retention and antibiotic therapy (DAIR), the intra-articular antibiotic infusion, and the use of methylene blue for biofilm eradication. Conclusions: the use of new cement spacers with xylitol, ammonium compounds, or silver nanoparticles is another promising technique to increase the eradication rate in two-stage revision. It is important for professionals to deeply understand the pathogenesis of PJI and the role of biofilms in its development in order to become familiar with these novel techniques that could reduce the burdens on healthcare systems.

## 1. Introduction

Periprosthetic joint infection (PJI) can be a catastrophic complication for both the patient and the healthcare system, with a prevalence ranging between 0.3 and 2.9% [1]. By 2030, an estimated two million total joint arthroplasties (TJAs) will be performed annually in the United States (US). With the current protocols, over 30,000 patients are expected to be diagnosed with PJIs each year, imposing an estimated USD 1.85 billion annual burden on the US healthcare system [2]. The main cause of the treatment failure is the biofilm presence on the implants and the surrounding tissues. The other problem is bacterial multi-resistance to antibiotics that is frequent nowadays, not only in hospital environments but also in extra-hospital environments.

Currently, the diagnosis of PJI is evolving towards the use of biomarkers. The International Consensus Group, in 2018, proposed the major criteria and minor criteria to confirm the diagnosis of PJI. A diagnosis of PJI should be established when at least one major criterion is met or when three out of five minor criteria are present. However, the consensus of authors emphasized that PJI may still be present even if these criteria are not fully met, particularly in cases involving less virulent organisms (e.g., *Cutibacterium acnes*) [3].

The conventional prevention of PJI consists of measures that can be divided into three groups: preoperative, intraoperative, and postoperative. The aim of preoperative prevention is the control of obesity, malnutrition, diabetes mellitus, smoking, bacterial colonization, etc. The intraoperative and postoperative measures that decrease the incidence of PJI are as follows: shaving of the surgical site, perioperative antibiotic prophylaxis, decolonization of the surgical site skin, intraoperative irrigation with antiseptic solution, wound closure and care, implant surface properties, the local release of antibiotics from cement, tranexamic acid administration, and the correction of anemia/hyperglycemia [1,4]. Despite accounting for all these factors, some patients will still develop PJI after surgery. Hence, there is a need for novel unconventional agents (non-antibiotic) and alternative methods of antibiotic application (beyond intravenous or oral routes) that can be used alone or as a complement to antibiotherapy or antibiotic prophylaxis.

We have reviewed the most significant topics on unconventional strategies for PJI prevention and treatment, including silver- and iodine-coated implants, the application of hydrogels, phage therapy, lysostaphin and antimicrobial peptides, the implementation of double DAIR, intra-articular antibiotic infusion, new spacers, calcium sulfate, and methylene blue for biofilm eradication.

## 2. Methodology

For each novel unconventional therapy, *in vitro* data, animal model data, and clinical data were requested. The animal and clinical data specifically focused on bone and joint infections. Additionally, the advantages and disadvantages of each treatment option were critically discussed. This systematic approach allows readers to better assess the potential clinical value of each novel therapeutic agent.

To ensure the comprehensive coverage of the relevant literature, a scoping review was conducted on PubMed Central^®^ articles published primarily between January 2010 and August 2024, using the following search terms: “No antibiotic treatment of PJI”, “Bacterial Biofilm Eradication Agents”, “Unconventional Prevention of PJI”, “Periprosthetic Joint Infection”, “PJI”, “Biofilm”, “Unconventional Treatment”, and “New Strategies”.

This narrative review aimed (1) to explore the clinically relevant concepts of PJI pathogenesis, (2) to identify the limitations and challenges in the current approaches to PJI treatment, and (3) to highlight promising strategies and developments that have the potential to improve the course of lower-extremity PJI. Ultimately, our goal was to support clinicians in overcoming the limitations of the current treatment methods by presenting a range of alternative, evidence-based options for future use.

## 3. Pathogenesis

During prosthetic implantation, bone and periprosthetic tissues become damaged, avascular, or necrotic. All these surgery-related events reduce the local concentration of the systemic antibiotics needed to prevent infection. The foreign body reaction after implantation increases the interstitial medium, which is a fibro-inflammatory zone with immunosuppression. This zone has an environment that is relatively inaccessible to the immune system due to the absence of normal blood irrigation to the periprosthetic tissue. This impairs the ability of polymorphonuclear neutrophils, lymphocytes and other cells, antibodies, and certain antibiotics to reach the implant surface for preventing or combating infection [1,4]. Therefore, any prosthesis is susceptible to infection not only during the perioperative period but also throughout its lifespan, requiring local antimicrobial protection over months or years [2].

## 4. Biofilm Eradication in PJI

The maturation state of the bacterial biofilm formed on the implant and the antimicrobial substances used for its eradication play a very important role in the success of PJI treatment [5]. In the early stages of biofilm formation, there is a higher probability of eradicating it with systemic antibiotic treatment combined with direct lavage. When the biofilm advances to a mature stage, the expectations for eradication are lower [6].

It is considered that the critical period in which one can eradicate the biofilm on the implant is approximately up to 4–6 weeks postoperatively [4]. After this period, the biofilm formed on the implant cannot be eradicated because of an exopolymer matrix (EPM) surrounding the microorganism that blocks the antibiotic treatment [4]. Moreover, the natural course of a PJI leads to the biofilm expanding to the bone–implant interface, producing bone sequestra and generating osteomyelitis, which in turn compromises the stability of the implant.

Many methods are being studied to interrupt biofilm formation or prevent its diffusion. Some strategies are based on material engineering such as anti-adhesive surfaces or antibacterial addition into the cement, over the prosthesis [7].

### 4.1. Quorum Sensing

**Figure 1:**
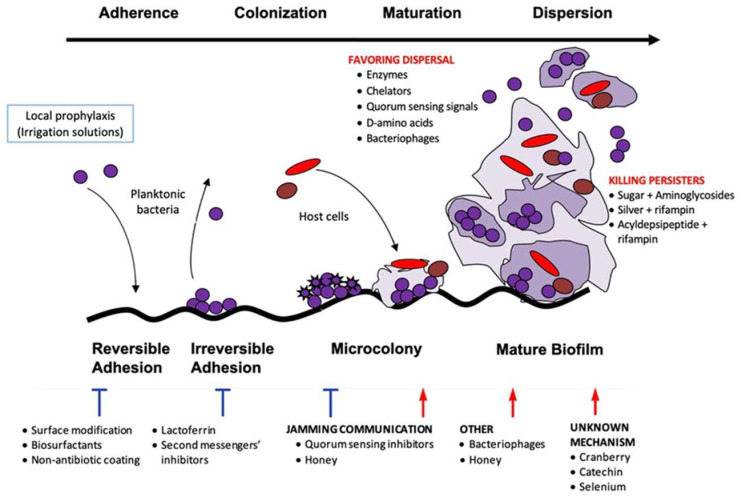
Stages of biofilm formation and anti-biofilm strategies.

Other ways are to act directly on the bacteria: inhibiting the quorum sensing (QS) detection system, preventing the formation of EPM, and inhibiting secondary messenger signaling pathways, among others [7]. QS comprises different enzymes produced by bacteria in the biofilm that allow the communication in between. The various strategies aimed at disrupting these signaling molecules among bacteria promote biofilm eradication (Figure 1, Table 1).

### 4.2. Material Topography

Regarding the material topography, it has been observed that the characteristics related to size, shape, and distribution of roughness patterns affect both the adhesion and biofilm formation of different bacterial strains on various substrates. Bacterial adhesion decreases as the topographic pattern size becomes smaller, and in this regard, micrometer-scale topographies mainly affect the bacterial attachment, while nanometer-scale topographies can have bactericidal effects [7]. However, this area of research is still in its early stages, and more studies are needed before these materials can be used.

### 4.3. Minimum Biofilm Eradication Concentration

Under normal conditions, in response to an implant infection, there is an immune response from the host with the proliferation of connective tissue over the metal surface, which significantly increases the resistance values of the biofilm to antibiotics compared to those obtained from *in vitro* measurements [8,9]. Typically, bacterial infections are treated with antibiotics doses that are above the Minimum Inhibitory Concentration (MIC). This concentration of the antibiotic will be bactericidal for planktonic bacteria but not for those in a biofilm. In traumatology, infected implants present a formed biofilm and require the management of the Minimum Biofilm Eradication Concentration (MBEC), which can be up to a thousand times higher than the MIC [4,6,10,11]. The challenging task of adequately measuring the MBEC, as there is no universally approved technique, is further complicated by reports that *in vitro* values do not match the *in vivo* MBEC values [8,9]. The *in vivo* MBEC depends on the implant surface, the environment, and the duration of exposure to antibiotics. However, the *in vitro* determination of the MBEC is currently the only parameter available to predict the treatment success, although cutoff points have not yet been established.

The literature review shows that this parameter has increasingly been studied and taken into account in recent years for testing antibiotics or various molecules against multiple microorganisms. Y. Okae et al. [9] studied the *in vitro* and *in vivo* MBEC for methicillin-sensitive *Staphylococcus aureus* (MSSA) and methicillin-resistant *Staphylococcus aureus* (MRSA). The study demonstrated a significant increase in the MBEC in an animal model compared to an *in vitro* model for the biofilm formed on a screw, due to the presence of fibroblasts, collagen, and cellular debris inhibiting proper antibiotic penetration to the implant. The maturation time was a crucial factor for biofilm eradication, with longer times correlating to higher MBEC values both *in vitro* and *in vivo*. High concentrations of local antibiotic therapy to eradicate the biofilm can be reduced via systemic treatment using antibiotics like rifampicin.

## 5. Unconventional Prevention Methods of PJI

### 5.1. Implant Surface Modification

The growing prevalence of antibiotic-resistant bacteria, fueled by the selective pressure from the widespread antibiotic use, globalization, and improper prescribing practices, poses a significant challenge to the development of effective preventive antibiotic therapies for various types of infections [12,13]. In this scenario, new non-antibiotic antimicrobials are gaining importance in the field of PJI prevention strategies. According to their strategy of action, implant surfaces with antibacterial properties can be classified into three groups: passive surface modification (contact killing), active surface modification (local elimination via the release of antimicrobials), and local antimicrobial carriers or coatings (Table 2). The destruction of bacteria can be achieved by interfering with respiration or cell division, cell wall formation, or bacterial signaling networks, as well as by inhibiting the transition from the planktonic phenotype to a sessile type [13]. The antibacterial surface technologies can employ metals (silver, zinc, copper, etc.), non-metallic elements (e.g., iodine, selenium), organic substances (antibiotics, anti-infective peptides, chitosan, other substances), and their combinations. Only a few antibacterial coating technologies are currently available for clinical use in traumatology. These include the following: silver and iodine coatings, antibiotic-loaded cement, gentamicin and polylactic acid coatings, and rapidly resorbable hydrogel coatings composed of covalently bonded hyaluronic acid and polylactic acid [14].

There are different strategies to incorporate heavy metals into titanium surfaces. The main heavy metals used to provide titanium alloys with antimicrobial capacity are silver, copper, and gallium. The types of surface modifications used to incorporate the metal into titanium surfaces are mainly the following: metallurgical addition, co-sputtering, ion implantation and coatings.

Regarding the use of these metal-based titanium alloy surface modifications in patients, it is notable that there are no comparative or prospective studies, only retrospective case series analyses. Only silver has been approved for use in humans and has demonstrated a reduction in the infection risk in studies with clinical cases. After analyzing the requirements of the antibacterial coating strategy that the “ideal” implant should possess, we would like to highlight the following: the safety (not systemic and local toxicity), large spectrum of bactericidal and antibiofilm activity *in vitro* and *in vivo*, resistance to press-fit insertion and to have acceptable cost [13]. It is possible that the use of metals for PJI prevention is just beginning, so new promising metallic candidates with antimicrobial capacity have not yet been employed. This includes metals such as nickel, cerium, selenium, cesium, yttrium, palladium, or superparamagnetic iron nanoparticles [12].

After analyzing the metal-based strategies, we should highlight their main advantages like the following: broad spectrum antimicrobial effect, the action against microorganisms directly adhered to the surface and those nearby, long durability, and the evidence in clinical trials that support their use. The main disadvantage is the local and systemic toxicity reported by some studies [12]. Metals exhibit a greater number of specific targets within bacteria compared to antibiotics. This characteristic is directly related to a reduced, but not nonexistent, occurrence of resistance to metals. These targets are attacked by metal cations and/or reactive oxygen species generated by both cations and metal oxides [12].

#### 5.1.1. *Silver*

Silver is the most widely used metal in biomedical applications. Dissolved silver cations are biochemically active agents that interfere with the bacterial cell membrane permeability and cellular metabolism. Silver also contributes to the formation of reactive oxygen species and other mechanisms that potentially affect prokaryotic cells [13]. Placing prostheses coated with silver metal particles in selected patients (immunocompromised patients, patients with osteoarticular oncology or chronic osteomyelitis, patients with a history of septic arthritis) significantly reduces the postoperative infection rates. These silver particles are slowly released into the adjacent tissues over an extended period, inhibiting the adherence of planktonic bacteria to the implant. A retrospective study by H. Wafa et al. [15] analyzed the results of using silver-coated tumor prostheses in primary surgeries and two-stage revisions, demonstrating a 48% reduction in the infection rates compared to conventional tumor prostheses. A clinical study conducted by D. Zajonz et al. [16] reported on 34 patients treated with modular mega-endoprosthesis after a cured bone infection of the femur or tibia. One group, consisting of 14 patients, received non-silver-coated implants, while the other group of 20 patients received silver-coated prostheses. During the 72-month follow-up period, the reinfection rate was 40% in the silver-coated group and 57% in the non-silver-coated group. However, due to the low number of cases, no statistical significance could be determined. Another study published by A. Piccioli et al. [17] analyzed the incidence of infection in patients who underwent surgery with silver-coated and non-coated tumor prostheses due to pathological limb fractures. In the silver-coated group, 11.8% of the patients developed an infection, compared to 23.1% in the non-coated prosthesis group. Additionally, no cases of early infection were observed in the silver-coated group, whereas early infection occurred in 66.7% of patients in the non-coated prosthesis group. A retrospective analysis by F. Donati et al. [18] examined 68 patients who underwent limb salvage surgery for various diagnoses, including osteosarcoma, Ewing sarcoma, chondrosarcoma, malignant fibrous histiocytoma of the bone, giant cell tumor of the bone, and metastasis. In the silver-coated implant group, the early infection rate was 7.9%, compared to 16.7% in the uncoated prosthesis group. Specifically, for early infection cases, the infection rate was 2.6% in the silver-coated group vs 10% in the non-coated group. A recent systematic review and meta-analysis conducted by H.I. Bulut et al. [19] compared the effectiveness of silver-coated mega prostheses in relation to titanium-coated implants. After analyzing 11 final studies comprising a total of 1419 patients, the reported incidence of PJI was 9.2% in the silver-coated group compared to 13.4% in the titanium-coated group. This finding underscores a significant difference in PJI incidence between the two groups, with a lower proportion of infections associated with silver-coated implants. These results highlight the potential superiority of silver coatings in reducing the risk of postoperative infections.

The routine use of silver-coated implants remains limited due to the potential cytotoxic effects of silver ions and the selective coating of prostheses, as the intramedullary part and some modular components cannot be coated [14]. The development of argyria (silver intoxication), whether local or systemic, has been poorly studied. The study by M. Glehr et al. [20] analyzed the incidence of local argyria in patients with silver-coated megaprostheses and its association with elevated silver levels both locally and in the bloodstream. Seven patients (23%) developed local argyria after a median of 25.7 months. Patients with and without local argyria had comparable levels of silver in their blood and aspiration fluids. Patients with local argyria had no elevated parameters for kidney or liver function. Additionally, cost issues and the inability to apply the technology to a variety of implants and prosthetic devices further reduce its application outside of oncological or highly selected cases.

#### 5.1.2. *Iodine*

Povidone-iodine, used as an electrolyte, forms a porous anodic adhesive oxide with the antiseptic properties of iodine. Preclinical and clinical studies have shown excellent results with the use of iodine-coated titanium implants. In the *in vivo* study conducted by K. Ueoka et al. [21], biofilm formation on different implant surfaces was compared, showing that iodine-coated titanium implants exhibited antibacterial activity for up to 8 weeks in rats. In a continuous, non-comparative series, H. Tsuchiya et al. [22] included patients diagnosed with bone tumors, limb deformities, osteomyelitis, pseudarthrosis, and fractures, all of whom underwent surgery using iodine-coated implants. The implants utilized included spinal instrumentation, osteosynthesis plates, pins and wires for external fixators, tumor hip and knee prostheses, nails, and cannulated screws. The infection rate was 1.9% with a follow-up of up to 18 months. No adverse effects have been reported to date. Nevertheless, there is still questionable long-term toxicity and challenging large-scale application of iodine-coated implants.

### 5.2. Hydrogels Loaded with Antimicrobial Agents

Antibacterial hydrogel contains hyaluronic acid grafted to polylactic acid (PLA) that is applied directly during surgery onto implants or tissues, protecting them from bacterial adhesion. It has been shown that hyaluronic acid has anti-adhesive properties when coating a metal implant, which reduces the impact of biofilm-related infections. It is used in combination with PLA to prevent bacterial adhesion, colonization, and biofilm formation due to its physical properties.

The hydrogel demonstrated synergistic antibacterial activity with various antibiotics and antibiofilm agents *in vitro* [23], and it can be used on all types of surfaces including titanium, nickel–chromium, chrome–cobalt, stainless steel, hydroxyapatite, and polyethylene [14]. It proved effective in a rabbit model of highly contaminated implants, both with and without systemic prophylaxis [24]. In a prospective multicenter randomized study, patients treated with a hydrogel applied to a plate and screw for osteosynthesis were compared to a control group. With a mean follow-up period of 18 months, the infection rate was 4.6% in the control group, whereas no infections were reported in the hydrogel-coated group [25].

A new promising strategy addressed to eradicate early PJI is Photothermal-AA gel. It contains D-amino acids known for their ability to disrupt biofilms, and pegylated gold nanoparticles, which facilitate thermoresponsive action. According to N. B. Milbrandt et al. [26], the application of the gel could induce localized hyperthermia, targeting the biofilm while minimizing harm to the surrounding tissues. In the study performed by C. A. Higuera-Rueda et al. [27], a rabbit model was utilized to test the anti-biofilm efficacy and safety of Phototherm AA in knee PJI treatment, finding it to be highly effective when used in conjunction with DAIR.

Despite the promising results, the long-term efficacy of the hydrogel, as well as its impact on implant osseointegration, requires further investigation.

## 6. Unconventional Treatment Methods of PJI

### 6.1. A Two-Stage DAIR

**Figure 2:**
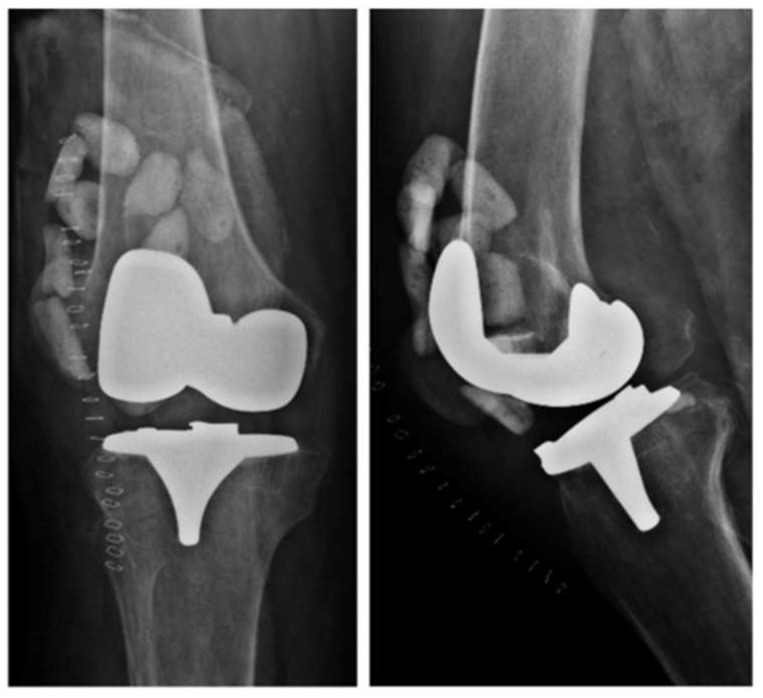
X-rays of a right knee with an infected total knee arthroplasty showing cement beads inside the joint capsule.

Double DAIR is a technique that, according to the literature, has a higher success rate than single DAIR [28]. After the debridement, irrigation, and replacement of the modular component, high-dose antibiotic-loaded cement beads (vancomycin 3 g/tobramycin 3.6 g) are left in place (Figure 2). Another lavage is performed after 7–14 days, during which the cement beads are removed. The study conducted by A. S. Chung et al. [28] demonstrated that two-stage DAIR is more effective than single-stage DAIR, achieving a success rate of 86.7% in acute PJIs of the hip and knee. Another clinical study by C. S. Estes et al. [29] reported a 90% success rate in patients treated with double DAIR.

On the other hand, B. A. Perez et al. [30] reported that the success rate of two-stage DAIR may be comparable to that of one-stage DAIR. Furthermore, the success of DAIR is well known as double DAIR procedures can be predicted using scores such as CRIME-80 and KLIC, which reflect the patient-related factors influencing the development of PJI [31]. Also, non-patient-related factors contributing to one-stage DAIR and two-stage DAIR failure include the presence of a biofilm on the implant, bacterial virulence, and the initial bacterial inoculum size [4]. Considering all these items, it can be concluded that neither double DAIR nor single DAIR will be effective treatments for certain patients.

### 6.2. Methylene Blue

**Figure 3:**
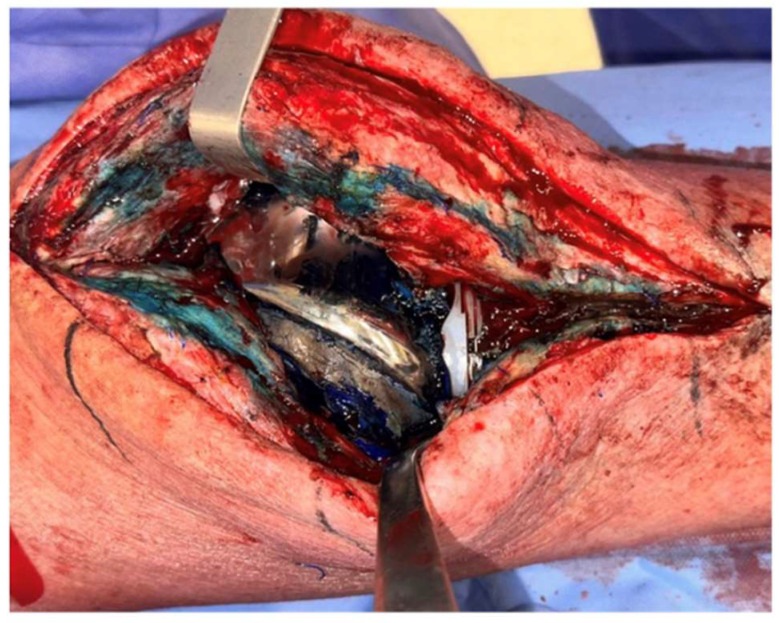
Clinical image of a medial parapatellar arthrotomy with prior injection of methylene blue into the knee joint. Notice the more pronounced staining in areas where there is more compromised tissue.

Methylene blue (MB) is a dye that selectively stains dead eukaryotic cells and bacteria. Prior to skin incision and arthrotomy, 50 mL of dilute 0.1% MB is then injected into the knee joint. The presence of a biofilm on the implant or at the tissue level, particularly in hard-to-reach areas, will specifically highlight its presence, helping the surgeon to distinguish it from unaffected tissues (Figure 3).

An *in vitro* study conducted by J. A. Parry et al. [32] demonstrated the strong staining capacity of MB on polyethylene liners, polymethylmethacrylate (PMMA), and teflon discs with biofilm growth. A nonrandomized prospective cohort study performed by J. D. Shaw et al. [33] compared the tissue samples of patients with PJI stained with MB vs unstained tissue. Across all the patient samples, significantly more bacteria were detected in MB-stained tissue compared to unstained tissue, as evidenced by a semiquantitative culture. Additionally, the tissue pathology revealed a higher number of polymorphonuclear leukocytes per high-power field in MB-stained tissue than in unstained tissue. The rate of infectious failure was 25%. During the procedures for secondary infection, all the cases had a microbiological profile that differed from cultures obtained during the MB stage of the study.

However, based on our experience, the use of MB can be challenging, as it often fails to clearly distinguish between dead and live tissues or biofilm.

### 6.3. Intra-Articular Antibiotic Infusion via Catheter

The delivery of antibiotics to the site of infection through cement elution or systemic routes is limited. In veterinary medicine, the practice of direct antibiotic injection for the treatment of purulent arthritis has been known for decades. In humans, intra-articular infusion with a Hickman catheter increases the local antibiotic concentrations by hundreds or even thousands of times compared to intravenous delivery while simultaneously minimizing the systemic toxicity. This technique is of great interest, especially for patients with resistant microorganisms (MRSA), chronic infections, and previous failures of two-stage revisions. Its significant advantage is the possibility of re-implanting the prosthesis in one stage and performing continuous intra-articular infusions for six weeks [34].

It is clear that intra-articular antibiotic infusion is only an adjuvant therapy for PJI, and it achieves better infection eradication rates compared to its use as a stand-alone treatment. The results of its use as an adjunct to DAIR surgery appeared superior when compared to studies utilizing DAIR alone. The failure rate for single-stage revision surgery with adjuvant intra-articular antibiotic therapy is 10%, compared to 21.2% for two-stage revision and 5.6% for DAIR procedures [35]. Notably, this DAIR failure rate is lower than the 10–31% failure rates reported in other studies and reviews for simple DAIR procedures [36,37,38,39,40].

However, the lack of statistical analysis and the relatively small subgroup populations limit the strength of the conclusions that can be drawn [35]. The drawbacks of this technique is an increased risk of superinfection with resistant bacteria or fungi, its high costs, and some discomfort for patients. Also, there are some studies reporting that there is no significant difference in the infection eradication rate using this method [41,42,43,44,45,46]. Additionally, some concerns have been raised about the development of fistulas and/or sinus tracts [41].

### 6.4. Phage Therapy

Bacteriophages or phages (viruses infecting bacteria) are the most abundant organisms on the Earth. Phages have various possible life cycles which, along with the interaction with their physical environment, dictate their role in bacterial ecology, diversity, and evolution. Although most bacteriophage life cycles are ascribed as being either lytic or lysogenic, the pseudolysogeny, carrier state, and chronic infection have been observed among different phage groups and in divergent environments [47]. Lytic phages are considered the most promising for incorporation as therapeutic tools for infection management. They cause bacterial lysis, the invasion of new bacterial cells, and continuous multiplication until complete bacteria elimination with low or null risk of resistance, unlike various antibiotics; thus, they can be used to treat multiple antibiotic-resistant infections [48,49,50,51]. Phage therapy is not used yet universally, as it is not yet an approved treatment by the EMA or FDA; rather, its use is limited to compassionate cases (FDA approved) or tolerated under the Declaration of Helsinki (still compassionate use). However, in some countries, such as Georgia and Russia, they are widely indicated for patients with recurrent PJI caused by resistant bacteria, particularly in situations where revision surgery is problematic or when patients refuse other options, such as amputations [52].

Certain bacteriophages act specifically against metabolically dormant bacteria, also called persister cells, as well as enzymatically degrading the biofilm matrix through the use of endolysins and depolymerases [53]. Additionally, the genetic engineering of phages can enhance their therapeutic potential by producing host range mutants via tail fiber mutations, exclusively lytic phages from lysogenic ones, non-toxic phages through gene deletions, and diagnostic phages by incorporating reporter genes [50,51,54,55].

The study conducted by C. Yilmaz et al. [56] developed a biofilm-associated osteomyelitis model in rats using methicillin-resistant *S. aureus* and *P. aeruginosa*. The research demonstrated a synergistic effect between phages and antibiotics, resulting in a significant reduction in the biofilm infection burden caused by both organisms. Another *in vitro* study performed by E. M. Ryan et al. [57] demonstrated that increasing the phage dosage from 10^4^ to 10^7^ PFU/mL significantly reduced the MBEC of cefotaxime from 256 mg/mL to 32 mg/mL in *Escherichia coli* biofilms. Phage therapy was investigated in an *in vivo* PJI model by S. Kaur et al. [58]. The author noted that coating the implant with phage and antibiotics provided the best therapeutic results to protect the implant against infection.

There remains a lack of clinical studies with a substantial number of patients treated with bacteriophages. The only nonrandomized, prospective study conducted by E. Fedorov et al. [59] reported 22 cases of combined phage–antibiotic therapy for hip PJI. All the patients underwent one-stage revision arthroplasty with intra-articular phage administration for 10 days. During the 12-month follow-up period, only one case showed persistent signs of infection, whereas the control group experienced eight cases of relapse. Additionally, J. B. Doub et al. [60]. reported a case series of 10 patients with chronic, recalcitrant knee, and/or hip PJI treated with adjuvant bacteriophage therapy—six cases underwent DAIR, and four underwent one- or two-stage revision surgery. Almost all the patients received combined intra-articular and intravenous bacteriophage therapy. Notably, none of the patients experienced the recurrence of PJI caused by the original infecting organism. The use of Defensive Antibacterial Coating (DAC^®^) hydrogel as a carrier for a phage cocktail was reported by T. Ferry et al. [61]. This approach, combined with other measures, was employed to address a recurrent knee megaprosthesis infection caused by *S. aureus*.

However, phage therapy has limitations, including the need for precise knowledge of the etiological agent before treating the patient, as each bacteriophage is specific to a narrow group of bacteria, precluding empirical treatment. Lytic bacteriophages only inhibit bacterial growth, necessitating concurrent appropriate antibiotic treatment. Bacteria can develop resistance to bacteriophages. As foreign agents, the body can develop immunity against bacteriophages, neutralizing them with antibodies. The pharmacokinetics and pharmacodynamics of phage therapy are complex and are not yet fully understood [50,51]. Regarding the side effects, there have been reports of elevated transaminases, steatosis, and hepatomegaly, highlighting the need for careful monitoring of liver function [62].

### 6.5. Lysostaphin

Lysostaphin is a metalloenzyme antimicrobial produced by *Staphylococcus simulans* biovar staphylolyticus. It has demonstrated bactericidal activity against MRSA strains and vancomycin-resistant *S. aureus*. Additionally, the activity of lysostaphin was independently tested against the biofilm of *S. aureus*, altering its EPM. There are multiple therapeutic applications of lysostaphin: nasal decolonization in MRSA carriers and application in infected wounds in the form of a hydrogel with chitosan. Good results have been demonstrated with its application in cement for the treatment of bone defects *in vitro* and *in vivo* [63,64]. Plates coated with lysostaphin have been used for the treatment of bacteria-contaminated fractures, showing better bone consolidation results with a less inflammatory response [65].

There are currently no clinical trials testing the therapeutic efficacy of lysostaphin on patients with PJI. All *in vivo* studies conducted so far have focused on nasal decolonization or the treatment of *S. aureus* skin and skin structure infections [65].

One of the problems with its use is immunogenicity: the immune system recognizes it as a foreign agent and neutralizes it with antibodies. Additionally, its widespread clinical application awaits the standardization of drug formulations, whether used alone or in combination with other antibiotics.

### 6.6. Antimicrobial Peptides

Due to the major problem of bacterial resistance in antibiotic treatment, new generations of antimicrobial agents with limited resistance are needed. Antimicrobial peptides (AMPs) are emerging agents for the treatment of PJI due to their broad-spectrum antibacterial properties, controllable biocompatibility, and diverse structures and categories. Compared to traditional antibiotics, AMPs rarely induce antimicrobial resistance due to their multiple antibacterial modes of action and their attacks on low-affinity targets, such as bacterial membranes, rather than a highly specific molecule. Different AMPs can have different properties: bactericidal, antifungal, antiviral, antiparasitic, immunomodulatory, and regenerative activities. The mechanisms of action on microorganisms depend on different physicochemical properties: charge, structure, sequence length, peptide concentration, hydrophobicity, and membrane composition. Due to their amphipathic properties, AMPs easily integrate into the cell membrane or pass through it to the cytosol. The antimicrobial mode of action can be classified into two types: on one hand, the integrity of bacterial structures such as the cell membrane and cell wall can be directly altered by peptides; on the other hand, antimicrobial peptides could directly induce the inhibition of cellular processes by binding to specific molecules. Most peptides exert their antimicrobial effects by destabilizing the negatively charged bacterial membrane due to anionic lipids, such as lipopolysaccharides for Gram-negative bacteria or teichoic acids for Gram-positive bacteria. Additionally, AMPs exhibit anti-biofilm activity across all stages of biofilm development through various pathways of action: inhibiting adherence, development, or stimulating dispersion [66,67].

An *in vitro* study conducted by P. Melicherčík et al. [68] has shown very promising results of bone cement loaded with AMPs to prevent microbial adhesion, as well as subsequent biofilm formation on its surface. A study of animal models has demonstrated promising results in the treatment of osteomyelitis using peptide-coated titanium plates in rabbits with infections following the internal fixation of open fractures [69]. Antimicrobial effects and favorable osseointegration were observed in peptide-loaded PEEK (poly-ether-ether-ketone) scaffolds implanted into infected rabbit femurs [70]. Furthermore, a study using mice revealed that scaffolds loaded with AMPs significantly promoted the osteogenic differentiation of mesenchymal stem cells and enhanced peri-implant bone formation [71]. There are currently no clinical studies evaluating the therapeutic efficacy of AMPs in patients with PJI.

Despite the numerous benefits of AMPs for bone and implant-related infections, several issues still need to be addressed. There is also a lack of toxicology studies that influence the development of AMPs, as well as a sparsity of clinical data that often translates into myriad regulatory issues [72]. Additionally, the high cost of production presents a significant barrier to scaling up AMP production and bringing them to market.

### 6.7. Quaternary Ammonium-Based Biomedical Materials

Quaternary ammonium compounds (QACs) are cationic surfactants and antimicrobials with a broad spectrum of activities. QACs act against planktonic forms of Gram-positive and Gram-negative bacteria, fungi, parasites, and some viruses, but they are not considered sporicidal. The antimicrobial mechanism of QACs is the disruption of the cell membrane, and the length of the N-alkyl chain affects their antimicrobial activities. The optimum chain length of QACs is 14 carbons for Gram-positive bacteria, 16 carbons for Gram-negative bacteria, and 12 carbons for yeast and filamentous fungi [73,74].

The use of quaternary ammonium-based biomedical materials and nanoparticles and the combination of QACs and nanomaterials are very promising for combating PJI. Quaternary amine dimethacrylate-modified bone cement showed contact-killing antimicrobial properties preventing biofilm formation without releasing any bioactive agents [73]. Modified bone cement with some QACs exhibits improved physical properties and better osteogenic activity than gentamicin-loaded PMMA [73]. In a rabbit model, hydroxypropyltrimethyl ammonium chloride chitosan-loaded PMMA demonstrated the inhibition of bone infections induced by methicillin-resistant *S. epidermidis* [75,76]. At the same time, there are studies showing that a titanium implant coating with QACs prevents bacteria attachment *in vitro*, decreases the infection rates associated with orthopedic implantation *in vivo*, and promotes implant osseointegration [77]. However, clinical trials and FDA approval are required before the clinical translation and commercialization of this technology.

The incorporation of both contact-based antimicrobials such as QACs and release-based antimicrobials such as silver nanoparticles produces surfaces with dual-functional antibacterial activities. Dual-functional coatings exhibit potent initial antibacterial properties due to the release of bioactive biocides and maintain long-term antibacterial activities. A high initial burst release offers protection against the initial elevated risk of infection, while sustained contact killing provides a defense against the subsequent latent infections [73].

However, there are growing concerns regarding the biocompatibility of QACs. Substantial evidence points to their potential toxicological side effects, which could hinder their safe and widespread application. A lower cytotoxicity also needs to be achieved to expand their clinical use.

### 6.8. New Spacers with Antibacterial Properties

In chronic PJI, the replacement of a prosthesis in one or two stages is inevitable. The new implant placed must be surrounded by a sterile environment; otherwise, a new failure will occur. One-stage exchange involves the aggressive debridement of the infected tissues, knowing the microorganism, its antibiotic sensitivity, and ensuring good soft tissue coverage. Two-stage exchange is the gold standard in the US. A cement spacer with antibiotics specific to the infecting microorganism is placed, slowly eluting into the surrounding tissues to ensure the complete eradication of the infection. The literature reports revision failure rates in one or two stages between 10 and 29%, without a clear advantage of one procedure over the other [2]. Studies examining the revision failure in two-stage PJI and the presence of a biofilm on the cement spacer during the second revision, when the definitive replantation is performed, demonstrate that antibiotic doses released from the cement over a long period are below the MBEC [32]. Subtherapeutic antibiotic concentrations allow the infection to persist, promoting bacterial survival, adaptation, and development in this less hostile environment for them [6]. For this reason, studies are exploring new strategies to eradicate chronic PJI by investigating new substances or antibiotic combinations that could maintain adequate concentrations over time.

**Figure 4:**
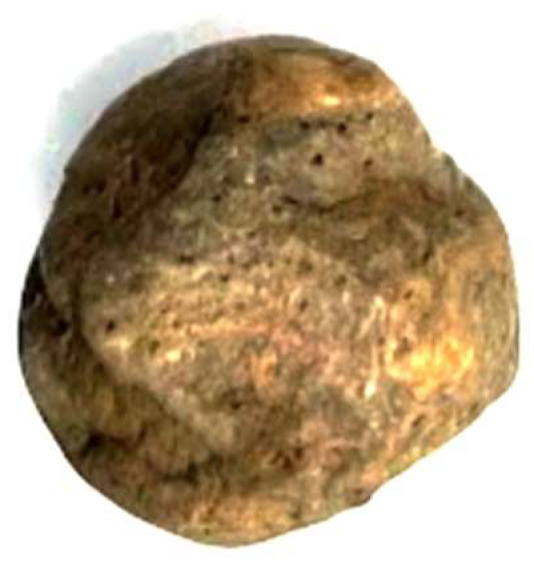
Cement bead with xylitol and silver nitrate. Note the presence of multiple micropores due to xylitol, which significantly increases the elution of substances contained in the cement.

Considering that only 10% of the antibiotic is eluted from the cement spacer in a two-stage revision, one way to increase this release would be by creating more porosity in the cement. Substances such as glycine and xylitol have long been known as pore formers in cement. The literature mentions a 40% release of the antibiotic mixed with cement when these molecules are added (Figure 4). Additionally, it has been demonstrated that xylitol has antibacterial properties and prevents the formation of a biofilm [33].

An *in vitro* study performed by J. Jackson et al. [78] demonstrated that the combination of silver nitrate with gentamicin, tobramycin, and vancomycin has a synergistic antibacterial action, which may prevent the development of antibiotic resistance, reduce antibiotic doses, and improve the spectrum of action to cover both Gram-positive and Gram-negative bacteria due to the non-specific bactericidal properties of silver. Other authors describe the use of copper spacers as an alternative to antibiotic-loaded cement. Copper is a metal with bactericidal and anti-biofilm activity. These metallic spacers are used in two-stage PJI treatment, although their use is still limited [79].

### 6.9. Calcium Sulfate Beads

Antibiotic-laden calcium sulfate (CS) beads are increasingly used in the treatment of PJI. Compared to PMMA beads, CS beads offer several advantages: they are bioabsorbable, demonstrate improved antibiotic elution characteristics, and have lower peak polymerization temperatures. Additionally, the ability to produce and store antibiotic beads for future use has the potential to standardize dosing, reduce the operating room time, and lower the healthcare costs [80].

**Figure 5:**
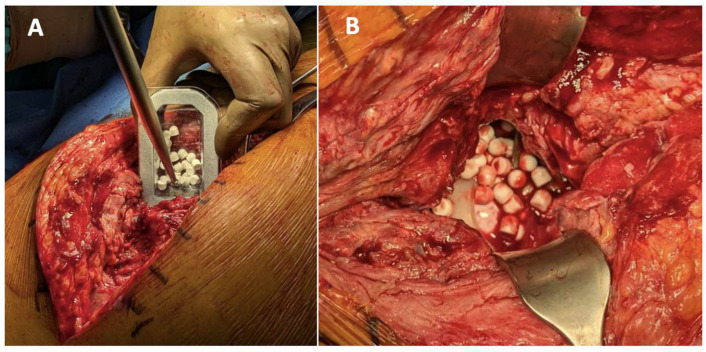
(**A**): An example of the use of CS with hydroxyapatite for PJI. (**B**): The beads are placed under the fascia, above the prosthesis or the spacer.

Antibiotic-loaded CS beads can be used both for PJI prophylaxis in primary arthroplasty and for its treatment (Figure 5). *In vitro* studies have shown promising results in reducing the biofilm formation by bacteria such as *Pseudomonas aeruginosa*, *Staphylococcus aureus*, and *Enterococcus* spp., with antibiotic retention lasting up to 38 days [81,82,83]. Some *in vitro* studies suggest combining antibiotic-loaded spacers with CS-loaded beads for improved clinical outcomes [84]. Additionally, *in vitro* and *in vivo* studies indicate that the local elution of vancomycin can reach concentrations up to 3000 times the MIC for MRSA. [84,85]

Several clinical studies have reported higher infection-free success rates for DAIR when using CS beads compared to DAIR alone. In the study conducted by J. C. V. Lachica et al. [86], two cohorts of total knee arthroplasty patients were compared—one group receiving prophylactic CS beads and the other without. Acute PJI was observed in 27 patients (67.5%) in the control group vs only 4 patients (9.3%) in the CS bead group. Another study performed by S. Ghirardelli et al. [87] reported an acute and hematogenous PJI eradication success rate with DAIR and CS antibiotic-added beads in 80% of the patients without comorbidities.

**Figure 6:**
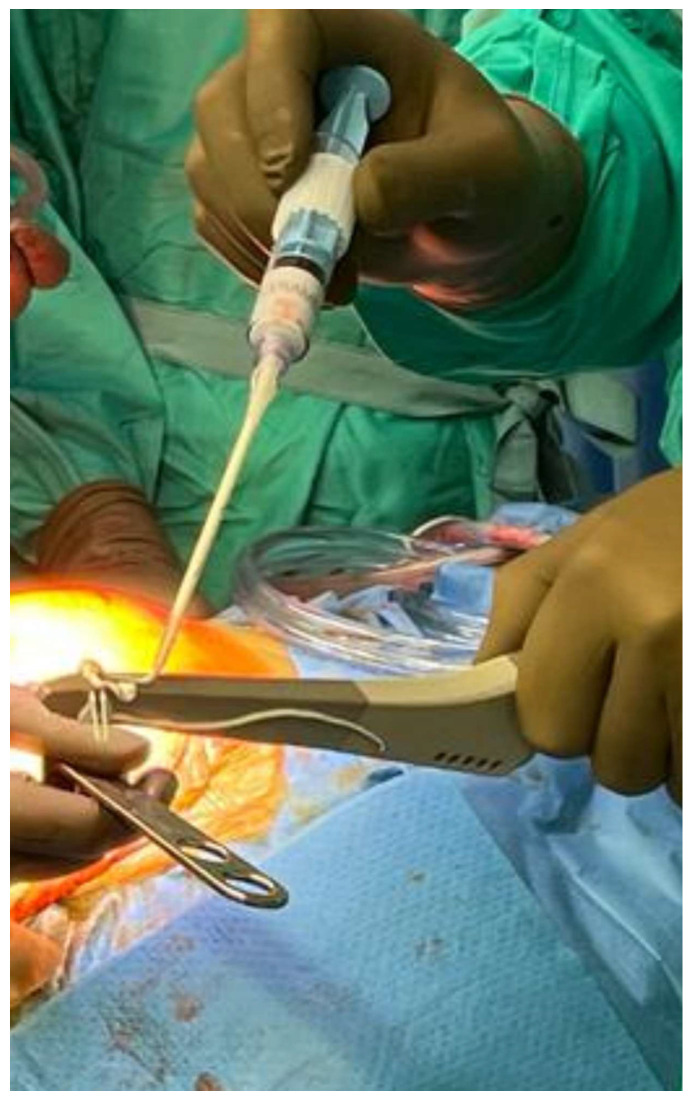
An example of using CS with hydroxyapatite (CERAMENT^®^) during the second stage of hip PJI revision. The uncemented stem is coated with CERAMENT^®^ prior to reimplantation.

The combination of CS with hydroxyapatite (e.g., CERAMENT^®^) has been implemented for chronic osteomyelitis, as well as acute and chronic PJI [88] (Figure 6). CS acts as a resorbable carrier, while hydroxyapatite is highly osteoconductive, promoting bone ingrowth [88]. Chronic osteomyelitis can persist due to the presence of dead and avascular bone, which harbors bacteria that invade the osteocyte lacuno-canalicular network of live cortical bone—contributing to persistent PJI [89]. The addition of local antibiotics helps eradicate infections in areas where systemic antibiotics cannot reach due to a compromised vascularization. Simultaneous de novo bone formation is observed throughout the material, and full remodeling into host bone occurs within 6–12 months. This rapid bone remodeling reduces the risk of fracture, non-union, and reinfection. Positive results have been reported using CS/hydroxyapatite as a bone graft substitute to restore acetabular bone loss in hip revision surgeries [87]. Furthermore, this treatment is more patient friendly, as it allows surgeons to manage bone defects in a single-stage procedure, eliminating the need for multi-stage surgeries. Transitioning from multi-stage to single-stage procedures also helps hospitals optimize their healthcare resources and reduce the costs [89,90,91,92].

Despite its advantages, some studies have reported a low effectiveness of CS in eradicating acute PJI, with success rates around 50% [93,94]. According to D. De Meo et al. [95] no significant difference in success rates between DAIR alone and DAIR combined with CS was observed. Furthermore, reports of hypercalcemia following the use of antibiotic-eluting absorbable CS beads in revision arthroplasty for infection have raised concerns [96].

## 7. Limitations

This article analyzes the alternative methods of PJI treatment and prophylaxis in primary arthroplasties described in the literature that may be incorporated into daily practice in the future. While our review serves as a valuable resource for summarizing the unconventional strategies for the prevention and treatment of PJI, it has certain limitations. Firstly, as a narrative review, it does not follow a systematic literature review approach. However, the authors conducted a thorough review of the literature to highlight the general consensuses and discrepancies, drawing upon both their expertise and current research. Another limitation is that some of the most recent research developments may not be captured, given the time required to compile and publish a comprehensive analysis. Finally, although this review aims to provide an overview of the alternative PJI treatments, its broad scope may limit the depth of analysis for individual studies, potentially overlooking the nuanced details and complexities within the current research landscape.

## 8. Conclusions

Incorporating new methods of PJI treatment and using them for prophylaxis in primary arthroplasties pave the way for improving the current situation. Further *in vivo* studies in animal and human models are needed to demonstrate that these therapies are safe, effective, and economically feasible for patients. While some new therapies have already been approved for use in patients, they are currently applied only in select cases where conventional treatments have failed. Their indication could potentially be expanded to include patients at risk of developing PJI in primary arthroplasties or those with a poor prognosis for infection eradication in revision surgeries.

Future reviews should focus on conducting an in-depth analysis of intraosseous antibiotic application for prophylaxis, coating surfaces with mesoporous titanium, combining metal nanoparticles with antibiotics in bone cement, and developing new antimicrobial delivery systems using nanotubes. In the coming years, the fastest-growing and most patient-applicable antimicrobial technique is likely to be hydrogels loaded with antimicrobial agents, both for prophylaxis and for the treatment of PJI.

## Figures and Tables

**Table 1:** Summary of the main strategies to disrupt biofilm.

Targets	Description of Effects on the Biofilm
Matrix components	Matrix-degrading enzymes
Autoinducers of quorum sensing system	Quorum-sensing suppressor enzymes or quorum-sensing inhibitors act by inactivating acyl-homoserine lactone molecules
Second messengers	Small organic molecules capable of inhibiting secondary messenger signaling pathways
Enzymes	Small-molecule inhibitors may be able to block SrtA by disrupting protein attachment
Incorporation of antibacterial agents	TiO_2_, SiO_2_, ZnO, AgVO_3_, fluorapatite, quaternary ammonium resin monomer, silver nanoparticles, fluorohydroxyapatite, and others. Most are bactericides and act via contact

Note. Adapted from [7].

**Table 2:** Examples of different antibacterial implant protection strategies.

Strategy	Feature	Examples
**Passive surface modifications**	Prevention of bacterial adhesion without releasing bactericidal agents	Hydrophilic surface, super-hydrophobic surface, anti-adhesive polymers, nano-patterned surface Albumin Hydrogels Biosurfactants
**Active surface modifications**	Active pharmacological agents:	
	• Inorganic	Silver ions and nanoparticles Other metals (copper, zinc, titanium dioxide, etc.) Non-metals: iodine Other non-metal ions (selenium, grapheme, etc.)
	• Organic	Coated/linked antibiotic Covalently linked antibiotic Antimicrobial peptides Cytokines Enzymes and biofilm-disrupting agents Chitosan derivatives
	• Synthetic	Non-antibiotic antimicrobial compounds
**Peri-operative antibacterial local carriers or coatings**	• Not-biodegradable	Antibiotic-loaded polymethylmethacrylate
	• Biodegradable	Antibiotic-loaded bone grafts and substitutes
		Fast-resorbable hydrogel

Note. Adapted from [13].
